# Supplementary material for: The genome sequence of pale flax, Linum bienne Mill. (Malpighiales: Linaceae)
Source: Wellcome Open Res. 2026 Sep 21;11:721. [Version 1] doi: 10.12688/wellcomeopenres.27876.1 (PMC13768704; doi:10.12688/wellcomeopenres.27876.1)
Supplement: Peer review history

# Reviewer report: Reviewer response for version 1

**DOI:** 10.21956/wellcomeopenres.30673.r178511

**Published:** 2026-11-13

**Authors:** Olawole O. Obembe

**Affiliations:** 1 Covenant University, Ota, Ogun State, Nigeria

**REVIEWER'S REPORT**

**Title:** *The genome sequence of pale flax, Linum bienne Mill. (Malpighiales: Linaceae)*

**General Assessment**

 The manuscript describes the haplotype-resolved genome assemblies of *Linum bienne* Mill. (pale flax), as part of the Darwin Tree of Life Project, which aims to generate high-quality reference genomes for all named eukaryotic species in Britain and Ireland.

 Overall, the study provides a useful genomic resource for this species. The sequencing, assembly and chromosome-level validation data are well described. Overall, the manuscript is technically sound and provides valuable genomic information. There are only a few points to clarify in this manuscript, mainly relating to the gene prediction methods.

**MAJOR COMMENTS**

**1. Genome size**

 The genome size was estimated using k-mer and flow cytometry analyses, producing estimates of 533.92 and 620.00Mb, respectively. The assembled genome was 486.84Mb across 41 scaffolds, representing haplotype 1, the reference genome for this species. The heterozygosity and repeat percentage were found to be 0.22% and 50.87%, respectively. This provides useful information regarding this species’ genome.

**2. Sequencing**

 The sequencing technology (PacBio HiFi and Hi-C), amount of sequencing data (21.74Gb & 155.74Gb) and number of reads (1.49 million reads & 515.69 million read pairs) have been well described. The genome coverage was found to be 79x (NCBI assembly), providing a substantial sequencing depth for the assembly.

**3. Genome assembly methodology**

 The authors provided sufficient details of the genome assembly pipeline in Table 1, including the tools and procedures employed.

**4. Assessment of assembly quality and completeness**

 The assembly statistics presented in Table 2 are comprehensive and include information on the number of gaps (334) and scaffold N50 (31.91 Mb).

 The reported quality metrics for haplotype 1, including 95.1% complete BUSCOs (22.4% single-copy and 72.7% duplicated), combined k-mer completeness of 99% and consensus quality (QV) of 66.7, indicate a high level of sequence completeness and base-level accuracy. Nevertheless, the high proportion of duplicated BUSCOs should be briefly explained in relation to the haplotype-resolved assembly strategy.

**5. Chromosome-level validation**

 The Hi-C contact map is well presented and provides supporting evidence for chromosome-level scaffolding. The lengths and GC content of all 15 chromosome-level pseudomolecules are also reported, contributing to the characterization of the assembled genome.

**6. Repeat annotation and gene prediction**

 The manuscript reports 68,357 transcripts, comprising 37,444 protein-coding genes and 18,570 non-coding genes. Although the authors have provided a link to the Ensembl annotation webpage, a brief description of the gene prediction and annotation methodology, including the principal software tools and supporting evidence employed, would improve the methodological transparency and reproducibility of the study. The authors may retain the Ensembl webpage link for access to further annotation details.

**7. Data availability**

 The accession numbers for the raw sequencing reads and final genome assembly are provided, which facilitates access to the genomic resource and supports future research.

**OVERALL RECOMMENDATION**

**Approved, subject to a minor revision.**

 The manuscript presents a useful and adequately characterised haplotype-resolved genome assembly of *Linum bienne*. The sequencing strategy, assembly statistics, quality assessment and chromosome-level validation provide reasonable support for the reported genomic resource.

 I recommend that the authors provide a description of the gene prediction and annotation methodology, including the software and supporting evidence employed, before indexing. A brief clarification of the high proportion of duplicated BUSCOs would also improve the interpretation of the assembly quality metrics.

 Subject to these minor clarifications, the manuscript is suitable for indexing.

Are the datasets clearly presented in a usable and accessible format, and the assembly and annotation available in an appropriate subject-specific repository?

Yes

Are sufficient details of the sequencing and extraction, software used, and materials provided to allow replication by others?

Yes

Are the rationale for sequencing the genome and the species significance clearly described?

Yes

Are the protocols appropriate and is the work technically sound?

Yes

Reviewer Expertise:

genomics, transcriptomics, genetic diversity studies, speed breeding, plant cell, tissue and organ culture, plant molecular farming, plant molecular breeding

I confirm that I have read this submission and believe that I have an appropriate level of expertise to confirm that it is of an acceptable scientific standard.

# Reviewer report: Reviewer response for version 1

**Published:** 2026-09-21

**Authors:** Automated Benchmarking

**Affiliations:** 1 Wellcome Sanger Institute, Hinxton, UK

As this article is a Genome Note, Automated Benchmarking has been used to determine whether the reported assembly passes four defined checks established by the Earth BioGenome Project: the raw and assembled data are publicly available, the Earth BioGenome Project three-part metric is met, the k-mer completeness exceeds 90%, and the BUSCO score passes the stated threshold. For Linum bienne (ddLinBien1), all four checks passed. Data available: pass. ENA study PRJEB76769, raw-read accessions ERR13304154 and ERR13317830, and assembly accessions GCA_965366145.1 and GCA_965366135.1 are public. Earth BioGenome Project three-part metric: pass. Haplotype 1 is reported as 6.C.Q67, meeting the 6.C.Q40 reference standard. k-mer completeness: pass. The combined haplotype assemblies have 99.07% k-mer completeness, exceeding the Earth BioGenome Project target of 90%. BUSCO: pass. Haplotype 1, which is proposed as the reference assembly for this species, has 95.1% complete BUSCOs (22.4% single-copy and 72.7% duplicated). BUSCO software version: 5.7.1; lineage dataset: eudicots_odb10. It meets the plant-specific target of greater than 90% complete BUSCOs. This decision uses values reported in the Genome Note and public accession records.

Reviewer Expertise:

NA

I confirm that I have read this submission and believe that I have an appropriate level of expertise to confirm that it is of an acceptable scientific standard.
